# Indices of insulin sensitivity and secretion from a standard liquid meal test in subjects with type 2 diabetes, impaired or normal fasting glucose

**DOI:** 10.1186/1475-2891-8-22

**Published:** 2009-05-28

**Authors:** Kevin C Maki, James M McKenney, Mildred V Farmer, Matthew S Reeves, Mary R Dicklin

**Affiliations:** 1 Provident Clinical Research, Bloomington, IN, USA; 2 National Clinical Research, Richmond, VA, USA; 3 Meridien Research, St Petersburg, FL, USA

## Abstract

**Background:**

To provide an initial evaluation of insulin sensitivity and secretion indices derived from a standard liquid meal tolerance test protocol in subjects with normal (NFG), impaired fasting glucose (IFG) or type 2 diabetes mellitus.

**Methods:**

Areas under the curve (AUC) for glucose, insulin and C-peptide from pre-meal to 120 min after consumption of a liquid meal were calculated, as were homeostasis model assessments of insulin resistance (HOMA2-IR) and the Matsuda index of insulin sensitivity.

**Results:**

Subjects with NFG (n = 19), IFG (n = 19), and diabetes (n = 35) had mean ± SEM HOMA2-IR values of 1.0 ± 0.1, 1.6 ± 0.2 and 2.5 ± 0.3 and Matsuda insulin sensitivity index values of 15.6 ± 2.0, 8.8 ± 1.2 and 6.0 ± 0.6, respectively. The log-transformed values for these variables were highly correlated overall and within each fasting glucose category (r = -0.91 to -0.94, all p < 0.001). Values for the product of the insulin/glucose AUC ratio and the Matsuda index, an indicator of the ability of the pancreas to match insulin secretion to the degree of insulin resistance, were 995.6 ± 80.7 (NFG), 684.0 ± 57.3 (IFG) and 188.3 ± 16.1 (diabetes) and discriminated significantly between fasting glucose categories (p < 0.001 for each comparison).

**Conclusion:**

These results provide initial evidence to support the usefulness of a standard liquid meal tolerance test for evaluation of insulin secretion and sensitivity in clinical and population studies.

## Background

Both impaired insulin sensitivity and pancreatic beta-cell dysfunction play central roles in the pathogenesis of type 2 diabetes mellitus. Methods for assessment of these parameters in research settings, including mathematical modeling of data obtained during intravenous glucose tolerance tests, as well as euglycemic and hyperglycemic clamp procedures, have been evaluated extensively [1-3]. However, the cost and sophistication of these methods generally restrict their use to studies evaluating a limited number of individuals.

A variety of methods of deriving surrogate measures of insulin sensitivity and secretion from the oral glucose tolerance test have been evaluated. These have been shown to perform reasonably well for discrimination between groups and individuals with differing levels of insulin resistance and beta-cell dysfunction [3-6]. Recently, Retnakaran *et al*. [7] demonstrated a hyperbolic relationship between the Matsuda index of insulin sensitivity and the ratio of the total areas under the curve (AUC) for insulin and glucose (AUC_ins/glu_) over 120 min derived from oral glucose tolerance tests in subjects with normal and impaired glucose tolerance. This is similar to the hyperbolic relationship between the acute insulin response to an intravenous glucose load and insulin sensitivity derived from the minimal model that has been demonstrated by Kahn et al. [8,9]. Thus, the product of these two indices (Matsuda index * AUC_ins/glu_) may be a useful tool for assessing beta-cell function in relation to the level of insulin sensitivity in population and clinical studies, analogous to the disposition index from the intravenous glucose tolerance test with minimal model analysis (acute insulin response * insulin sensitivity index).

In recent years, the roles of incretins released postprandially (e.g., glucagon like peptide-1 and gastric inhibitory peptide) as modulators of glucose homeostasis and appetite have become topics of intense interest [10-12]. Incretin responses vary according to glucose tolerance status and may differ depending on the nature of the stimulus. For example, Vollmer *et al*. [12] found that the gastric inhibitory peptide response to a mixed meal was 186% larger than that elicited by an oral glucose load.

In addition, among individuals at increased risk for development of diabetes, multiple metabolic and hemodynamic disturbances (i.e., the cardiometabolic syndrome) are often present. In clinical and epidemiological studies it may therefore be of interest to assess not only determinants of glucose homeostasis, but also other metabolic variables such as postprandial lipid, lipoprotein and incretin responses. Accordingly, the ability to assess insulin sensitivity and secretion from a meal tolerance test has substantial utility for epidemiologic and clinical research, and has been evaluated in several studies [12-18]. However, the meals employed have typically utilized solid foods, which may present problems regarding standardization across multiple research sites due to varying ingredients used in food preparation from region to region and country to country; differences in meal preparation methods; and subject-related elements such as the degree to which foods are chewed. The aim of this investigation was to provide an initial assessment of insulin sensitivity and secretion indices using a widely available and easily standardized liquid meal tolerance test in subjects with normal fasting glucose (NFG), impaired fasting glucose (IFG) or type 2 diabetes.

## Methods

The data for the present investigation are from tests completed for the control condition in a clinical trial. Data were collected from three clinical research sites in the United States under Good Clinical Practice Guidelines, the Declaration of Helsinki (2000), and the US 21 Code of Federal Regulations (Part 50 – Protection of Human Subjects). An institutional review board (Schulman Associates Institutional Review Board, Inc., Cincinnati, OH) approved the protocol before the initiation of the study. Informed consent was obtained from all subjects before beginning any protocol-specific procedures. Subjects were informed of their right to withdraw from the study at any time.

### Subjects

Men and non-pregnant, non-lactating women with and without type 2 diabetes were enrolled. Eligible participants were each 18–74 y of age with body mass index <40 kg/m^2^. Subjects without diabetes were required to have no history of glucose intolerance or signs or symptoms of hypoglycemia and to have screening levels of glycosylated hemoglobin (HbA_1c_) ≤6.0%, and fasting plasma glucose <6.11 mmol/L. Subjects with diabetes had been diagnosed at least one year earlier and treated for at least 12 weeks with stable dose(s) of a sulfonylurea alone or with a sulfonylurea plus metformin and/or a thiazolidinedione, and had screening levels of HbA_1c _≤9.0% and fasting plasma glucose <11.1 mmol/L. Individuals with poorly controlled hypertension or significant renal, pulmonary, hepatic or biliary disease; a recent history of a cardiovascular event; or any gastrointestinal condition that could potentially interfere with absorption of the study product were excluded. The use of selected medications within eight weeks of the initiation of screening procedures was not allowed, including insulin, meglitinides, alpha-glucosidase inhibitor agent(s) and systemic corticosteroids.

### Test Meals

Subjects received standard meal tolerance tests (active and control) on different days ~1 week apart in a crossover design. Only data from the control test are presented. Subjects were instructed to consume a diet containing at least 150 g/d of carbohydrate and to avoid vigorous physical activity during the 48 h prior to the meal test, confirmed by queries when subjects arrived for testing after fasting for at least 10 hours. Subjects consumed half of a 502 g, 500 kcal chocolate flavored shake (Ensure^®^, Abbott Nutrition, Abbott Laboratories, Columbus, OH), ingested two placebo capsules, and then consumed the second half of the shake over a 10 min period. The shake contained 80 g carbohydrate, 12 g fat, and 18 g protein. Any medications normally taken by the subject in the morning were administered 20 min prior to the start of the test meal.

### Blood Pressure

Using an automatic blood pressure monitor (Vital Signs Monitor 300 Series, Welch Allyn^®^, Beaverton, OR), duplicate systolic and diastolic blood pressures were measured after at least 5 min of seated rest and the two values averaged.

### Laboratory Measurements

Blood samples were collected from an indwelling catheter for an assessment of the fasting lipid profile and measurements of glucose, insulin and C-peptide pre-meal (average of two values at times = -5 and 0 min) and 30, 60, 90, 120, 150, 180, 210, and 240 min after the beginning of test meal consumption. Samples were drawn into chilled, heparinized tubes and centrifuged rapidly to avoid glycolysis. Biochemical measurements were completed by Medpace Reference Laboratory (Cincinnati, OH). Plasma glucose was measured by photometry following a hexokinase reaction [19]. Plasma insulin [20] and C-peptide [21] were analyzed using electrochemiluminescence immunoassays.

### Statistical Analyses

Statistical analyses were generated using SAS version 9.1.3 or Statview version 5.0 (SAS Institute, Cary, NC). All tests of statistical significance were completed at the 5% level. The analysis dataset included all subjects who completed a control test and had complete data for glucose and insulin values pre-test and for the first 120 min post-meal. Total AUCs for glucose, insulin and C-peptide from pre-meal to 120 min were calculated using the trapezoidal rule [22]. Homeostasis model assessments of insulin resistance (HOMA2-IR) and pancreatic beta-cell function (HOMA2-%B) were completed using the HOMA Calculator version 2.2.2 (, accessed July 2008). The Matsuda index of insulin sensitivity was calculated as follows, with glucose and insulin values in mmol/L and pmol/L, respectively [4]:



where G_0 _and I_0 _are pre-meal values for insulin and glucose and G_m _and I_m _are mean post-meal values during the first 120 min of the liquid meal tolerance test. The indices evaluated were selected *a priori *based on their performance in previous investigations [7,13], and the timepoints measured (0–120 min) were utilized because they are anticipated to have the greatest utility in large-scale population and multi-center clinical trials when a relatively short test is desirable.

Subjects without diabetes were categorized as having NFG if the average of their pre-meal plasma glucose values was <5.56 mmol/L or IFG if this average was ≥ 5.56 mmol/L [23]. Characteristics of the study participants according to fasting glucose tolerance categories (NFG, IFG, diabetes) were compared for continuous variables using analysis of variance, followed by the Scheffé test for pairwise comparisons. For categorical variables, chi-square tests were employed for comparisons between the three glucose tolerance categories and for pairwise comparisons between categories where the overall chi-square test showed statistical significance. Linear and non-linear regression analyses were used to evaluate relationships between indicators of insulin secretion and sensitivity.

## Results

### Subjects

Table 1 shows characteristics of the study sample, categorized by fasting glucose tolerance status, which included 73 subjects, 19 each with NFG and IFG and 35 with diabetes. Of the subjects with diabetes, 9 were using a sulfonylurea only, 16 were using a sulfonylurea plus metformin, 4 were using a sulfonylurea plus a thiazolidinedione and 6 were using all three classes of medication. The percentage of male subjects did not differ significantly for those with NFG (26.3%) and IFG (47.4%), but was higher for subjects with diabetes (80.0%) than in the other two fasting glucose tolerance categories. The percentage of subjects who were non-Hispanic white (83–84%) was similar, and not significantly different, in the three fasting glucose tolerance groups. Mean age was similar between those with NFG and IFG, but higher in subjects with diabetes. Body mass index was highest among subjects with diabetes, intermediate among those with IFG and lowest in subjects with NFG. Total and low-density lipoprotein cholesterol concentrations did not differ across categories, but high-density lipoprotein cholesterol was significantly lower in those with diabetes than in the other two groups. Mean fasting triglycerides and systolic blood pressure were significantly higher among subjects with diabetes compared with the NFG group, despite the fact that a greater percentage of subjects with diabetes were taking antihypertensive medication (4.3%, 18.2% and 59.0% in the NFG, IFG and diabetes groups, respectively). Diastolic blood pressure did not differ across fasting glucose tolerance categories. Mean glycosylated hemoglobin levels were in the normal range for subjects with NFG and IFG and <7.0% for those with diabetes, consistent with good glycemic control for a majority of the subjects with diabetes.

**Table 1:** Characteristics of study subjects categorized by fasting glucose tolerance status

				P-Values		
				
Parameter	NFG	IFG	Diabetes	NFG vs. IFG	NFG vs. Diabetes	IFG vs. Diabetes
Total Number, M/F (n/n)	19 (5/14)	19 (9/10)	35 (28/7)	0.313	< 0.001	0.031
Age (years)	45.1 ± 4.2	45.7 ± 3.3	61.0 ± 2.0	0.989	<0.001	0.002
BMI (kg/m^2^)	25.8 ± 1.0	29.3 ± 1.2	32.7 ± 0.7	0.071	< 0.001	0.045
Total cholesterol (mmol/L)	5.35 ± 0.39	5.17 ± 0.24	4.72 ± 0.17	0.903	0.202	0.437
LDL cholesterol (mmol/L)	3.19 ± 0.35	2.96 ± 0.18	2.69 ± 0.14	0.799	0.246	0.649
HDL cholesterol (mmol/L)	1.53 ± 0.09	1.44 ± 0.12	1.07 ± 0.05	0.758	< 0.001	0.005
Triglycerides (mmol/L)	1.38 ± 0.18	1.73 ± 0.27	2.29 ± 0.25	0.695	0.048	0.308
Systolic BP (mm Hg)	117.0 ± 3.4	122.6 ± 3.3	129.1 ± 2.3	0.484	0.015	0.284
Diastolic BP (mm Hg)	72.6 ± 1.9	78.2 ± 1.8	77.1 ± 1.6	0.165	0.214	0.918
Fasting glucose (mmol/L)	5.22 ± 0.05	5.87 ± 0.06	7.53 ± 0.29	0.249	< 0.001	< 0.001
Fasting insulin (pmol/L)	52.8 ± 6.0	82.0 ± 11.0	124.8 ± 13.9	0.373	< 0.001	0.068
Fasting C-peptide (nmol/L)	0.63 ± 0.05	0.89 ± 0.08	1.27 ± 0.08	0.110	< 0.001	0.004
HbA_1C _(%)	5.2 ± 0.1	5.2 ± 0.1	6.7 ± 0.1	0.907	< 0.001	< 0.001
Glucose AUC (h*mmol/L)	11.1 ± 0.5	14.1 ± 0.8	23.1 ± 0.8	0.059	< 0.001	< 0.001
Insulin AUC (h*pmol/L)	894 ± 121	1346 ± 180	922 ± 103	0.102	0.988	0.075
C-peptide AUC (h*nmol/L)	4.2 ± 0.3	5.6 ± 0.4	4.7 ± 0.3	0.032	0.531	0.162
AUC_ins/glu _(pmol/mmol)	78.6 ± 8.8	95.5 ± 12.2	40.7 ± 4.4	0.398	0.003	< 0.001
HOMA2-IR	1.00 ± 0.11	1.57 ± 0.21	2.48 ± 0.26	0.346	< 0.001	0.037
HOMA2-%B	83.7 ± 5.9	89.5 ± 7.9	86.7 ± 10.6	0.936	0.976	0.981
Matsuda Index	15.6 ± 2.0	8.8 ± 1.2	6.0 ± 0.6	0.003	< 0.001	0.254

Values are mean ± SEM unless otherwise specified.

Abbreviations: AUC = area under the curve, AUC_ins/glu_= ratio of the total areas under the curve for insulin and glucose, BMI = body mass index, BP = blood pressure, HbA_1c _= glycosylated hemoglobin, HDL = high-density lipoprotein, HOMA2-IR = homeostasis model assessment of insulin resistance, HOMA2-%B = homeostasis model assessment of pancreatic beta-cell function, IFG = impaired fasting glucose, LDL = low-density lipoprotein, M/F = male/female, NFG = normal fasting glucose, Diabetes = type 2 diabetes mellitus

### Fasting and Post-Meal Indices of Carbohydrate Metabolism

**Figure 1:**
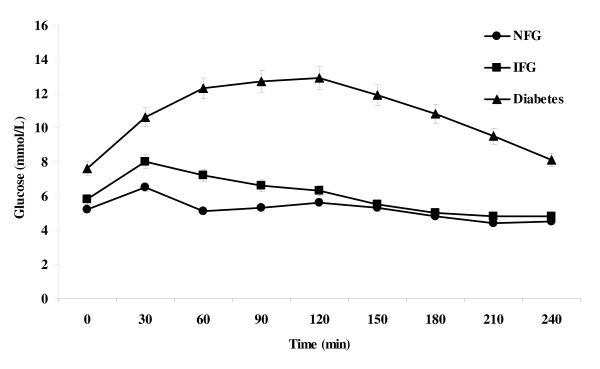
**Mean ± SEM plasma glucose values by timepoint during a standard liquid meal tolerance test according to fasting glucose tolerance status**. NFG = normal fasting glucose; IFG = impaired fasting glucose; Diabetes = type 2 diabetes mellitus.

**Figure 2:**
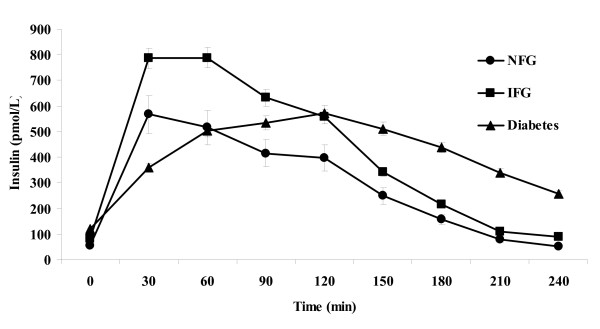
**Mean ± SEM plasma insulin values by timepoint during a standard liquid meal tolerance test according to fasting glucose tolerance status**. NFG = normal fasting glucose; IFG = impaired fasting glucose; Diabetes = type 2 diabetes mellitus.

Glucose and insulin responses during the standard liquid meal tolerance test among subjects in the three fasting glucose tolerance categories are shown in Figures 1 and 2. The AUC for glucose during the first 120 min of the test increased progressively from NFG to IFG to diabetes. Peak glucose and insulin responses occurred in the first 60 min among subjects with NFG and IFG, but at 120 min among subjects with diabetes. The AUC for insulin and C-peptide were numerically higher among subjects with IFG than among those with NFG, although the differences did not reach statistical significance. Subjects with diabetes had insulin and C-peptide responses that were intermediate between the NFG and IFG groups.

### Insulin Resistance

Insulin resistance, as indicated by the HOMA2-IR index or the inverse of the Matsuda insulin sensitivity index, was greatest among those with diabetes, intermediate among those with IFG and lowest among those with NFG. The difference between the diabetes and NFG groups was statistically significant by both measures (Table 1). Only the HOMA2-IR index differed significantly between subjects with IFG and diabetes, whereas only the Matsuda index differed between subjects with NFG and IFG.

### Beta-Cell Function

**Figure 3:**
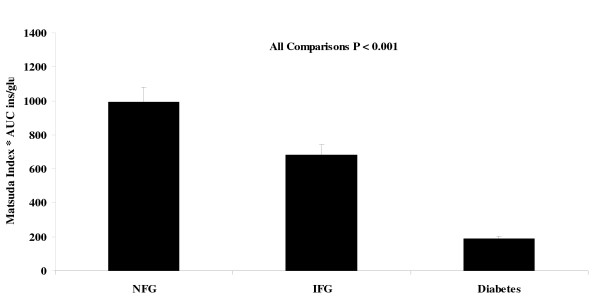
**Results for the product of the Matsuda index of insulin sensitivity and the ratio of the areas under the curve for insulin to glucose over 120 min (AUC ins/glu) according to fasting glucose tolerance status**. NFG = normal fasting glucose; IFG = impaired fasting glucose; Diabetes = type 2 diabetes mellitus.

Surprisingly, the HOMA2-%B value did not differ significantly across fasting glucose tolerance categories (Table 1). In contrast, the product of the AUC_ins/glu _and the Matsuda index showed highly significant differences for all comparisons (Figure 3). Compared to the mean value of 995.6 ± 80.7 in the NFG group, those with IFG showed a value that was 31% lower and those with diabetes had a mean value that was 81% lower.

### Correlations between Measures of Insulin Resistance and Beta-Cell Function

Table 2 shows Pearson correlation coefficients between the various measures of insulin resistance (or sensitivity) and beta-cell function. Natural logarithmic transformations improved the fit for some variables. The Matsuda index was strongly inversely correlated with HOMA2-IR, overall and in all three fasting glucose tolerance groups, with correlation coefficients of -0.914 to -0.942.

**Table 2:** Pearson correlation coefficients showing the relationships between indices of glucose homeostasis according to fasting glucose tolerance status

	Log_e _HOMA2-IR	Log_e_AUC_ins/glu_	HOMA2-%B
	Normal Fasting Glucose		
Log_e _Matsuda Index	-0.940†	-0.791†	-0.935†
Log_e _HOMA2-IR	1.000	0.684†	0.959†
Log_e _AUC_ins/glu_	--	1.000	0.699†
	Impaired Fasting Glucose		
Log_e _Matsuda Index	-0.917†	-0.766†	-0.826†
Log_e _HOMA2-IR	1.000	0.741†	0.946†
Log_e _AUC_ins/glu_	--	1.000	0.789†
	Type 2 Diabetes Mellitus		
Log_e _Matsuda Index	-0.914†	-0.745†	-0.338*
Log_e _HOMA2-IR	1.000	0.717†	0.491†
Log_e _AUC_ins/glu_	--	1.000	0.631†
	All Subjects		
Log_e _Matsuda Index	-0.942†	-0.237*	-0.418†
Log_e _HOMA2-IR	1.000	0.185	0.520†
Log_e _AUC_ins/glu_	--	1.000	0.511†

*P < 0.05; † P < 0.01

Abbreviations: AUC_ins/glu_= ratio of the total areas under the curve for insulin and glucose, HOMA2-IR = homeostasis model assessment of insulin resistance, HOMA2-%B = homeostasis model assessment of pancreatic beta-cell function

The Matsuda index and the HOMA2-IR index each showed similar strengths of correlation with AUC_ins/glu _within all three groups (r values of ± 0.684 to ± 0.791). The Matsuda index was much more strongly inversely correlated with HOMA2-%B in those with NFG (r = -0.935) and IFG (r = -0.826) than in those with diabetes (r = -0.338). Similarly, HOMA2-IR was strongly correlated with HOMA2-%B in the NFG (r = 0.959) and IFG (r = 0.946) groups, but the correlation was less strong for those with diabetes (r = 0.491).

### Relationship between Insulin Secretion and Sensitivity

**Figure 4:**
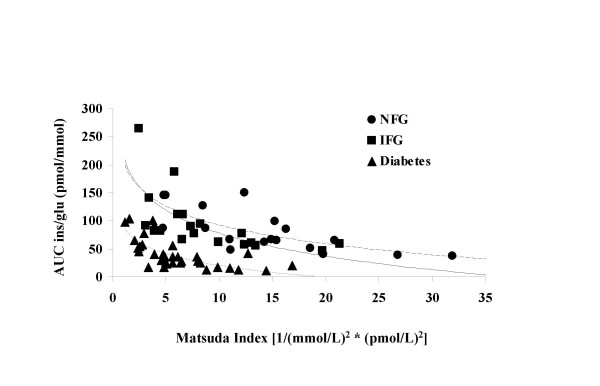
**Regression lines illustrating the relationships between the Matsuda index of insulin sensitivity and the ratio of the areas under the curve for insulin to glucose over 120 min (AUC ins/glu in pmol/mmol) according to fasting glucose tolerance status**. NFG = normal fasting glucose (Y = 380.365*X^-0.652^); IFG = impaired fasting glucose (Y = 258.174*X^-0.548^); Diabetes = type 2 diabetes mellitus (Y = 110.751*X^-0.734^) Key: Circle = NFG; Square = IFG; Triangle = Diabetes

The relationship between insulin secretion and insulin sensitivity can be expressed as insulin secretion = constant/insulin sensitivity [7-9]. The pattern observed is consistent with the expectation that declining glucose tolerance would be associated with shifts to the left and downward (Figure 4). However, it would also be expected that the regression lines would have exponent values equal to approximately -1.0 [equivalent to a slope of -1.0 in the equation log_e _AUC_ins/glu _= intercept * slope (log_e _Matsuda index)]. The slopes were all significantly different from zero, but were also significantly different from -1.0: NFG slope = -0.652 (95% CI -0.910 to -0.394), IFG slope = -0.548 (95% CI -0.784 to -0.313), diabetes slope = -0.734 (95% CI -0.967 to -0.502).

## Discussion

This investigation provides preliminary evidence suggesting that a standard liquid meal tolerance test may be used to assess indices of insulin sensitivity and secretion across the spectrum of glucose tolerance. The results conformed to the expected relationships between insulin secretion and sensitivity, as indicated by the AUC_ins/glu _and the Matsuda index, respectively. The product of these two indices, which is analogous to the disposition index (acute insulin response * insulin sensitivity index) described by Kahn *et al*. [8,9] was able to discriminate between the three categories of fasting glucose tolerance, even with relatively small numbers of subjects. This product may therefore provide a useful measure of the ability of the pancreas to match insulin secretion to the prevailing degree of insulin resistance.

Intravenous glucose tolerance tests and glycemic clamp procedures are not practical for many clinical and epidemiologic investigations because of the cost and high level of sophistication required to conduct such tests. The oral glucose tolerance test provides a possible alternative for use in such instances. However, a meal tolerance test has a number of potential advantages compared to the oral glucose tolerance test. Because insulin resistant states are associated not only with disturbances in carbohydrate metabolism, but also with fasting and postprandial lipid levels, it may be desirable to simultaneously assess post-load glucose and lipid responses, without the need for tests on separate days. A mixed meal tolerance test also provides a more physiologic stimulus for assessing incretin responses [12]. Some subjects experience nausea or reactive hypoglycemia after a load consisting of glucose only. Neither of these issues was experienced by any of the 73 subjects in our sample, although subjects with a history of signs or symptoms of hypoglycemia were excluded from participation. In addition, the liquid meal used in our study is a commercially available product that is easily reproducible and requires no food preparation, thus can be employed easily in a standardized fashion across multiple research sites.

Other investigators have studied liquid or solid mixed meal loads for assessing insulin secretion or sensitivity [12-18]. In particular, Aloulou and colleagues [13] evaluated the ability to estimate insulin sensitivity using a standard breakfast test with various methods, most of which were originally validated using oral glucose tolerance test data. Using the minimal model-derived insulin sensitivity index from intravenous glucose tolerance test results as their standard for comparison, they found that the Matsuda index was the strongest correlate of insulin sensitivity (r = 0.656, p < 0.001). Retnakaran *et al*. [7] also recently reported that the product of the Matsuda index and the AUC_ins/glu _was the best indicator of the appropriateness of insulin secretion for the level of insulin sensitivity among several measures they evaluated. These findings prompted the authors to select the Matsuda index and the AUC_ins/glu _as the two measures for evaluation in the present study. In addition, these indices can be easily calculated using a spreadsheet, without the need for sophisticated mathematical modeling software. The liquid test meal used in the present investigation was similar to those employed previously in studies of insulin secretagogue medications [15,24].

The current study did not have gold standard indices of insulin secretion or sensitivity against which to compare the liquid meal tolerance test derived indices. The strong inverse correlations between the HOMA2-IR and Matsuda indices in all three fasting glucose tolerance groups (r = -0.914 to -0.940) suggest that both are measuring the same underlying physiologic process. Means for both indices showed the expected order, in that subjects with diabetes had the greatest degree of insulin resistance, subjects with NFG had the least, and subjects with IFG had intermediate values. Some caution is warranted regarding the correlation between these measures because the HOMA2-IR value is based on the product of fasting insulin and glucose levels, whereas the Matsuda index contains this product as an element of the calculation. However, both have also been shown to correlate well with minimal model and euglycemic clamp assessments of insulin sensitivity [4,25].

Using a minimal model analysis of data from meal tolerance tests, Steil *et al*. [18] showed that the insulin sensitivity index from a meal tolerance test correlated with that from a euglycemic clamp (r = 0.760, p < 0.001) to a degree similar to that derived from an intravenous glucose tolerance test (r = 0.717, p = 0.001), but that the index derived from the meal tolerance test was consistently higher than that from the intravenous glucose tolerance test by a factor of ~2.3. These results are in agreement with those from Caumo and colleagues [14] who also found a strong correlation between insulin sensitivity indices derived from minimal model analyses of meal and intravenous glucose tolerance tests, but with 2.2-fold higher values from the meal tolerance test (Spearman r = 0.89, p < 0.01). Thus, the available data suggest that a meal tolerance test (liquid or solid) can provide an appropriate stimulus for assessing insulin sensitivity, although absolute values from such tests may not be directly comparable to those derived from the euglycemic clamp or intravenous glucose tolerance test.

The authors were surprised that HOMA2-%B did not discriminate between the three fasting glucose tolerance groups. The subjects with diabetes generally had good glycemic control as indicated by an average HbA_1c _concentration <7.0% and were all taking sulfonylureas, which may have contributed to higher values than might have been the case otherwise. However, very similar values were present in subjects with diabetes, IFG and NFG. In contrast, AUC_ins/glu _did discriminate between diabetes and the other categories, although not between the NFG and IFG subjects. The product of the Matsuda index and AUC_ins/glu _discriminated between all three groups, consistent with the view that greater insulin secretion was partially compensating for the somewhat higher level of insulin resistance present in the subjects with IFG, and underlining the fact that the appropriateness of the pancreatic beta-cell insulin response can only be interpreted in relation to the prevailing degree of insulin resistance. Recently, Festa and colleagues [26] reported that HOMA-%B underestimated the degree of beta-cell dysfunction in subjects with impaired glucose tolerance and early-stage, asymptomatic type 2 diabetes, thereby supporting our results and confirming that post-load measures of beta-cell function are more sensitive indicators of beta-cell dysfunction than those derived from fasting values.

The relationship between insulin sensitivity (Matsuda index) and insulin secretion (AUC_ins/glu_) in the present study was consistent with the expectation that progressive worsening of glucose tolerance status from NFG to IFG to diabetes would be associated with shifts downward and to the left in the regression curves. However, slope of the regression line for the equation log_e _AUC_ins/glu _= intercept * slope (log_e _Matsuda index) did not approximate -1.0 in any of the three fasting glucose tolerance groups, as would be expected based on results for the oral glucose tolerance test described by Retnakaran *et al*. [7]. Values for the slope of this regression line ranged from -0.734 to -0.548. The reason for this difference between our results from liquid meal tolerance tests and theirs from oral glucose tolerance tests is unclear and will need to be investigated further. As discussed above, findings from other investigators have suggested that insulin sensitivity indices from mixed meal tests correlate with those from methods that use an isolated glucose stimulus, but are not always directly comparable in absolute terms. One potential explanation for the apparent difference is that differences in incretin responses elicited by a mixed meal, compared with an oral or intravenous glucose stimulus, alter the Matsuda index to AUC_ins/glu _relationship [12].

The present investigation was intended as an initial evaluation of particular measures of insulin secretion (AUC_ins/glu_) and sensitivity (Matsuda index) derived from a standard liquid meal tolerance test. The results are encouraging, but should be considered only a first step. Additional studies will be needed to compare this approach directly to established methods such as minimal model analyses, as well as euglycemic and hyperglycemic clamp methods for assessing insulin sensitivity and secretion. Moreover, studies will be required to assess the test-retest coefficient of variation in order to facilitate sample size calculations for clinical intervention trials. While it can be argued that the use of systemic insulin concentration as a surrogate marker of beta-cell function was flawed because it reflects both pancreatic secretion and hepatic extraction, the objective of this trial was to assess the utility of methods previously evaluated using oral glucose or mixed solid meal tolerance test data, but substituting a mixed liquid meal as the stimulus. Those previous examinations collected pre- and post-load insulin and glucose data, and we followed the same approach.

Some additional limitations of the present study should also be considered. The sample was relatively small and selected for participation in a clinical trial. Subjects with diabetes had good average glycemic control and were all taking sulfonylurea medications. The subjects with IFG did not cover the full range of fasting glucose levels for this category due to the exclusion criteria established for participation in the trial. Because no oral glucose tolerance test was completed, it was not possible to categorize glucose tolerance in subjects without diabetes beyond the classifications of NFG and IFG. Several investigators have shown that subsets of subjects with pre-diabetes such as those with isolated IFG, isolated impaired glucose tolerance, or both, differ regarding the degree to which peripheral insulin resistance, hepatic insulin resistance and beta-cell dysfunction contribute to their type and degree of glucose intolerance [3,27-29]. In the present study, some of the subjects with NFG and IFG might have been shown to have impaired glucose tolerance if oral glucose tolerance test results had been available. Therefore, testing in a broader sample across the entire spectrum of fasting and post-load glucose tolerance will be needed to demonstrate the generalizability of our results. Simultaneous measurement of incretins and parameters related to lipid metabolism would help to establish the utility of the standard liquid meal tolerance test for evaluation of multiple metabolic responses.

## Conclusion

In conclusion, the results from the present study suggest that the standard liquid meal tolerance test may be a useful alternative for assessment of insulin secretion and sensitivity in clinical and population studies. Additional research is warranted to further evaluate the validity and reliability of this test for use in such settings.

## Competing interests

The authors declare that they received research support from Cargill Corporation (Wayzata, MN) and have provided consulting to Abbott Nutrition (Columbus, OH).

## Authors' contributions

KCM contributed to the conception and design of the study. All authors contributed to the generation, collection, assembly, analysis and/or interpretation of the data. KCM and MRD were involved in the drafting of the manuscript. All authors approved the final version of the manuscript.
